# Barriers to sight impairment certification in the UK: the example of a population with diabetes in East London

**DOI:** 10.1186/1471-2415-14-99

**Published:** 2014-08-15

**Authors:** Rabia Bourkiza, Mala Subash, Dania Qatarneh, Joanna Dacosta, Catey Bunce, Tunde Peto

**Affiliations:** 1 Moorfields Eye hospital NHS, 162 City Road, ECV1 2PD London, UK; 2 NIHR Biomedical Research Centre at Moorfields Eye Hospital NHS Foundation Trust and UCL Institute of Ophthalmology, 162 City Road, ECV1 2PD London, UK

**Keywords:** Diabetic retinopathy, Visual acuity, Sight impairment certification

## Abstract

**Background:**

This study assessed the barriers to sight impairment certification in the East London Borough of Tower Hamlets amongst patients attending the Diabetic Retinopathy Screening Service (DRSS).

**Methods:**

All patients who attended DRSS between 1^st^April 2009 and 31st of March 2010 and whose recorded best corrected visual acuity (BCVA) at DRSS fulfilled the requirements for sight impairment in the UK were included. An additional 24 patients whose general practitioners (GPs) reported them to be certified blind due to no perception of light (NPL) vision were re-examined to ascertain the reason for certification, and their potential social and visual aids needs.

**Results:**

78 patients were identified with certifiable vision and were reviewed: 10 deceased in the preceding 12 months; 60 were not known to be certified. Of these, 57 attended further assessment, 27 were found to have non-certifiable vision, 9 were referred for further interventions, 9 were certified and 9 were found to be eligible, but declined certification. Five patients were registered due to diabetic eye disease.

Of those 24 reported by the GP of NPL vision, only 4 had true NPL, the rest had usable vision. Only two of them were certified blind due to diabetes.

**Conclusions:**

Our data shows that sight certification in patients with diabetes might be underestimated and these patients often have non-diabetes related visual loss. We propose that data on certifiable visual impairment could serve, along with existing certification databases, as a resource for quality of care standards assessment and service provision for patients with diabetes.

## Background

Diabetic eye disease remains the second cause of blindness among middle age working adults in the UK [1], and is on the priority list of the Vision2020 initiative [2]. In England, the National Screening Committee (NSC) recommends photographic screening for diabetic retinopathy as its preferred model. The main aim of the National Diabetic Retinopathy Screening Service (DRSS) is to reduce incident blindness due to diabetic retinopathy [3].

Despite increasing efforts to detect and treat diabetic retinopathy at an early stage, the incidence of certified visual impairment doubled between 1991 and 2001 in people over 65 years of age [4]. However, recent reports show an overall decline in the number of blind and partially sighted certifications in England [5]. This might be attributed to the fact that patients with diabetes live longer but remain at risk of the complications of the disease.

Accurate data on blindness and sight certification is paramount in facilitating the delivery of social support to those who will benefit from it. It is also an important statistic for continual monitoring at a social, economic and public health level. Data collected on blindness certificates has however been subject to criticism [6,7]. It was estimated that over 50% of eligible patients were not certified sight-impaired despite being seen by an ophthalmologist. It is necessary that measures to address this underestimation of visual impairment are taken. For these measures to be objective and purposeful, the level and reasons for under-certification amongst patients with diabetes must be understood.

This study had the following aims:

1. To assess the agreement on certifiable visual acuity between DRSS and hospital clinics in the London Borough of Tower Hamlets.

2. To identify the proportion of certifiable patients who are certified, and reasons for non-certification.

3. To explore visual acuity data gathered as part of a previous audit to examine variability in visual function over time.

## Methods

Objectives 1 and 2 were addressed by analysing data from two cohorts of patients

Cohort one: All patients attending the Tower Hamlets Primary Care Trust DRSS between 1st April 2009 and 31st of March 2010 whose recorded best corrected visual acuity (BCVA) at DRSS fulfilled the requirements for sight impaired or severely sight impaired certification. Certifiable visual acuity at DRSS was defined as 6/24 or worse in the better eye. This decision was based on the criteria for sight impairment to include BCVA of 6/24 with moderate contraction of visual field, media opacities or aphakia.

A data query was run by Digital Healthcare on Optomize® to list all patients with this level of vision and then the patients were manually verified after a careful filtering process.

Once patients were identified as having certifiable visual acuity at DRSS, a case note review at Moorfields Eye Hospital NHS Foundation Trust and The Barts and the Royal London NHS Trust was conducted. Data collected included: date of birth, last date of clinic visit, visual acuity, grade of retinopathy and maculopathy at the last entry in the clinical notes (after the screening visit), ocular co-morbidities and sight impairment certification. Patients who had not attended the hospital since their last screening visit were invited to an assessment clinic to have their certification status verified and to be offered an appointment in the low vision clinic if needed. Patients who did not attend this clinic were offered a further hospital appointment and failing attendance of this were invited to a screening appointment and a review by the clinical lead. The patients were given a maximum of 4 appointments to have their vision checked and their registration status verified.

Following the extra clinical assessment by the clinical lead, patients were offered sight impaired or severely sight impaired certification if appropriate. If patients did not wish to be certified, reason for this was recorded. Patients with reversible visual loss secondary to cataract or refractive errors were referred to the appropriate services.

Those already certified and those certified during the clinic visits had the reason for certification identified if possible.

Cohort Two: The medical records of patients who had been excluded from the DRSS due to bilateral no perception of light (NPL) vision as confirmed by their general practitioner were reviewed. This second cohort was to serve as quality control for the current clinical practice. The clinical notes were scrutinized for recorded BCVA and severely-sight impaired certification. All patients who were identified as having better than NPL vision were invited to attend a hospital appointment. All patients were given at least two appointments at clinic, and then two additional appointments at the screening service to be reviewed by the clinical lead. For those who did not attend, the relevant GPs were contacted to confirm NPL vision and certification status.

The Tower Hamlets Primary Care Trust Low Vision Team helped to determine certification status for some of the patients.

Objective 3 was addressed by examination of data gathered in a previous audit [8] (methodology described in Bunce C et al.) which collected visual acuity data as recorded in hospital records on every occasion that the patient attended. This audit consisted of 16 patients with diabetic disease and scatter plots of visual acuity against date of visit for each patient were constructed. There were 9 female and 7 male patients. The median age was 67 years with an interquartile range of (59, 76) years.

The audit was approved by Moorfields Eye Hospital NHS Foundation Trust audit committee. The audit adheres to the tenets of the Declaration of Helsinki, and followed the full code of ethics with respect to subject recruitment, subject testing and data protection. The audit forms part of the National Screening Committee’s rolling audits as well as having been approved by the Moorfields Eye Hospital Audit Committee, Patients attending diabetic eye screening service are subjected to continuous audits to ensure data quality, and as such no additional written consent was required.

## Results

Cohort 1: Of the 12137 patients registered on the Tower Hamlets DRSS database (of whom 7234 screened during the study period), 78 patients with certifiable vision were reviewed. Of these, 8 (10.2%) patients were already certified. Five patients had unknown status, 55 patients were not certified, and 10 patients died between screening and the clinic review which took place 12 months after screening.

Of the 60 patients not known to be certified, 57 were invited for an additional clinic or screening appointment to determine their certification eligibility and status due to prior failed attendance (n = 19), unavailability of notes (n = 25), awaiting intervention (n = 3) e.g. cataract surgery, or due to being initially identified as certifiable but not certified (n = 10). The remaining 3 had their status confirmed from the notes. The outcome of the clinic is shown in Table 1.

**Table 1:** Outcome of patients invited to the assessment clinic

**Outcome of assessment clinic**	**Number of patients (n = 60) including the 3 patients with notes review only**
Already certified (but not known from the database or the notes)	1
Not certified as vision found to be non-certifiable	27
Not certified as referred for further investigations or surgery	9
Certified	9
Did not attend to any of the 4 appointments provided	7
Certifiable but declined certification, cataract surgery or glasses	7

Overall, out of the 78 patients referred from DRSS, 27 (34.6%) were found to have non-certifiable vision, 9 (11.5%) were already certified (8 identified initially from the database and 1 only after patient consultation in clinic), 9 (11.5%) were found to have certifiable vision and they were consequently certified, 9 (11.5%) had certifiable vision but were referred for further intervention in the form of cataract surgery, refraction or investigations, 7 with certifiable vision (8.9%) declined certification or further intervention, 10 (12.8%) deceased, and 7 (8.9%) did not attend their appointments despite several invitations.

There were 18 patients of this cohort certified by the end of the study. The reasons for certification as sight impaired or severely sight impaired were: Diabetes (5 patients, 27.7%), AMD (2 patients, 11.1%), other retinal disease (3 patients, 16.6%), congenital (1 patient, 5.5%), optic atrophy (1 patient, 5.5%), corneal disease (1 patient, 5.5%), not known (5 patients, 27.7%).

Cohort 2: comprised of 24 GP referrals with no perception of light (NPL). All of these patients were certified as severely sight impaired. Two patients died in the interim and after further assessment of the remaining 22, only 4 patients were found to have true NPL vision.

**Figure 1:**
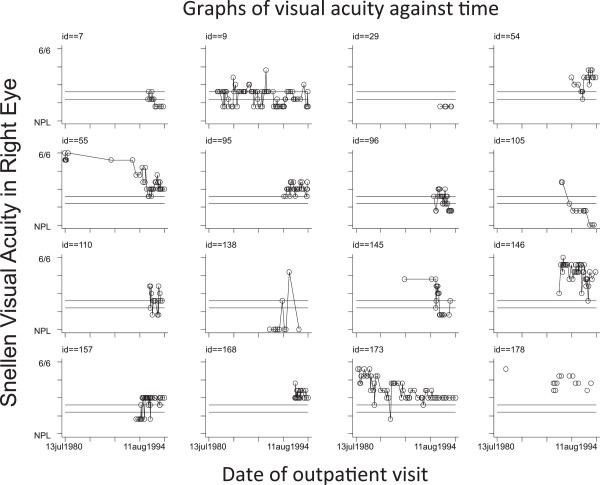
**Scatter plots of visual acuity against time.** Lines at 3/60 and 6/60 VA denote cut off for certification in patients with normal visual fields.

Amongst the 22 living patients in this cohort, the reasons for certification were: glaucoma (4 patients, 18.1%), optic atrophy (3 patients, 13.6%), AMD (3 patients, 13.6%), diabetes (2 patients, 9%), other retinal disease (2 patients, 9%), cataract (1 patient, 4.5%), corneal disease (1 patient, 4.5%), trauma (1 patient, 4.5%) and unknown cause in 5 patients (22.7%). The latest visual acuities ranged from 6/12 to Hand movements in the better eye.To illustrate the variability in visual function of diabetic patients, Figure 1 shows the scatter plots of visual acuity against date of visit for each of the 16 patients included in the previous audit.

## Discussion

The Royal College of Ophthalmologists and the National Screening Committee state reduction in sight certification as the aim of the DRSS [9,10]. Sight certification however, still relies on a manual register, which results in an underestimation. Margrain TH et al. reported that 14% of those with certifiable visual acuities were not certified and not known to social services [11]. It is interesting that the number of certificates in 2007–2008 as reported by Bunce et al. was significantly lower than that from 1999–2000 [12]. This decline was also reported in the latest data on sight certification in 2011 [5]. This fall in the number of certificates is surprising as epidemiological modelling and anecdotal data would suggest an increase. Bunce et al. acknowledged that many people who are eligible for certification may not be certified and that many people who are certified, may not always satisfy the criteria for certification [12]. While their data on sight certification should not be devalued, it’s important to have a more robust system of identifying the population of certifiable vision; those who require certification and those who opt out of certification. In our study, there have been a number of difficulties in identifying patient registration status from the hospital registration database, patients’ medical records and the electronic record system. Because of this, we elected to contact the patients directly to determine certification status and to invite those who were not certified to an extra clinic for further assessment.

A considerable number of patients are seen in the community screening programmes and not in hospital clinics and may have non-certified certifiable vision. Our study showed that visual acuity measured in the DRSS does not consistently match that in the hospital clinic. Interestingly, some patients who had certifiable vision at previous hospital appointments were subsequently found to have non-certifiable vision in the extra clinic. A few had had cataract surgery in the interim, others may have received treatment and differences might also be attributable to different methods of measuring vision (Snellen vs logMAR). The fluctuation, however, does need to be taken into consideration when assessing someone for certification [13], and clearly accurate measurement of vision is essential as differences may result in patients being denied potential benefits of certification or unnecessarily expose them to the distress that may accompany the offer of certification. Figure 1 illustrates the fluctuation in vision over time, which makes it difficult to determine when a patient is certifiable or not. A single measure of visual acuity should not be used to certify a patient.

Another factor that contributes to underestimation of sight impairment when looking at certification data is that certification is voluntary. Patients may not wish to be certified for various reasons including perceiving no significant benefit, lack of understanding or for cultural reasons where blindness may carry a stigma [14]. In our study 7 patients declined certification and when asked for the reason they attributed it to personal choice.

In addition, doctors may not fully appreciate the criteria for certification and this is consequently overlooked [6]. These factors may result in inadequate service evaluation and less targeted healthcare provision.

## Conclusions

We suggest that these barriers to certification (inaccuracy or discrepancy of visual assessment, manual certification process, patients and doctors’ understanding of certification, patients being seen in the community vs hospital) may be addressed and resolved with an electronic system. Software have recently been available to enable collection of data on new certifiable vision, and prompt the ophthalmologist to offer certification to the patient if indicated.

We propose that data on certifiable visual impairment could serve, along with existing certification databases, as a resource for quality standards assessment, research, and service provision for patients with diabetic eye disease.

## Competing interests

The authors declare that they have no competing interests.

## Authors’ contribution

TP conceived the study. RB, MS, DQ, JD and CB all contributed to the design of the study and collection of data. RB analysed the data and drafted the manuscript. All authors read and approved the final manuscript.

## Pre-publication history

The pre-publication history for this paper can be accessed here:

http://www.biomedcentral.com/1471-2415/14/99/prepub
